# Quantitative Analysis of Bioactive Compounds in Commercial Teas: Profiling Catechin Alkaloids, Phenolic Acids, and Flavonols Using Targeted Statistical Approaches

**DOI:** 10.3390/foods12163098

**Published:** 2023-08-17

**Authors:** Yuan Chen, Lingling Lai, Youli You, Ruizhen Gao, Jiaxin Xiang, Guojun Wang, Wenquan Yu

**Affiliations:** 1 Fujian Academy of Agricultural Sciences, Fuzhou 350003, China; chenyuan@faas.cn (Y.C.); 18639424217@163.com (R.G.); 19949533447@163.com (J.X.); 2 Fujian Tea Science Society, Fuzhou 350013, China; lily8011@sina.com; 3 Yongchun County Cultivation Service Center, Quanzhou 362699, China; ycnjz@163.com; 4 College of Horticulture, Fujian Agriculture and Forestry University, Fuzhou 350002, China; 5 Harbor Branch Oceanographic Institute, Florida Atlantic University, Fort Pierce, FL 34946, USA; guojunwang@fau.edu

**Keywords:** alkaloids, biomarker, catechins, flavonols, tea classification

## Abstract

Tea, an extensively consumed and globally popular beverage, has diverse chemical compositions that ascertain its quality and categorization. In this investigation, we formulated an analytical and quantification approach employing reversed-phase ultra-high-performance liquid chromatography (UHPLC) methodology coupled with diode-array detection (DAD) to precisely quantify 20 principal constituents within 121 tea samples spanning 6 distinct variants. The constituents include alkaloids, catechins, flavonols, and phenolic acids. Our findings delineate that the variances in chemical constitution across dissimilar tea types predominantly hinge upon the intricacies of their processing protocols. Notably, green and yellow teas evinced elevated concentrations of total chemical moieties vis à vis other tea classifications. Remarkably divergent levels of alkaloids, catechins, flavonols, and phenolic acids were ascertained among the disparate tea classifications. By leveraging random forest analysis, we ascertained gallocatechin, epigallocatechin gallate, and epicatechin gallate as pivotal biomarkers for effective tea classification within the principal cadre of tea catechins. Our outcomes distinctly underscore substantial dissimilarities in the specific compounds inherent to varying tea categories, as ascertained via the devised and duly validated approach. The implications of this compositional elucidation serve as a pertinent benchmark for the comprehensive assessment and classification of tea specimens.

## 1. Introduction

Tea, obtained from the freshly plucked leaves of *Camellia sinensis*, represents a universally consumed potable [1]. In China, the postharvest processing of *Camellia sinensis* leaves involves a series of six distinct techniques, leading to the production of six types of teas: black tea, green tea, yellow tea, white tea, oolong tea, and dark tea [2,3]. These processing methods have evolved over thousands of years across various regions of China and are generally classified into five categories based on the extent of endogenous enzymatic reactions: (1) non-fermented tea, such as green tea; (2) lightly fermented tea, including yellow tea and white tea; (3) partially fermented tea, represented by oolong tea; (4) fully fermented tea, exemplified by black tea; and (5) post-fermented tea, wherein exogenous microbial fermentation plays a crucial role in the processing. Figure S1 illustrates the distribution of tea types across China. Despite the extensive research conducted on tea and its chemical composition, flavor profiles, and health benefits, there is still much to explore and understand, particularly in relation to the unique chemical profiles of each tea type and their potential implications for human health. The variations in processing techniques and the involvement of different enzymatic reactions and microbial fermentation in tea production contribute to the unique characteristics and properties of each tea variety. Therefore, a comprehensive investigation into the chemical constituents and sensory attributes of these teas is crucial for establishing a deeper understanding of their distinct qualities and potential health implications.

Tea is replete with an array of chemical constituents, prominently inclusive of catechins, phenolic acids, flavonols, and alkaloids, all of which collectively constitute major bioactive constituents [4,5]. These components confer palatability and concurrently confer bioactivities such as antioxidation and antibacterial effects. Fermentation, primarily manifesting as enzymatic oxidation, orchestrates the conversion of tea polyphenols into the corresponding oxidation byproducts, which are eventually activated upon the exposure of tea leaves to ambient humidity and oxygen. The diverse chemical constituents and polyphenol oxidases evident within the six tea types stem from the varying extents of fermentation. Hence, a systematic and exhaustive evaluation of the constituents within the six tea varieties is of paramount significance. Noteworthy endeavors have been directed towards investigating functional constituents, notably catechins, purine alkaloids, and flavonol glycosides, within select Chinese tea variants [6,7,8]. Notably, tea polyphenols, particularly catechins, undergo oxidative transformations during manufacturing processes, instigated by either moist heat or intrinsic polyphenol oxidases alongside microbial oxidases [9]. Consequently, non-fermented green tea characteristically boasts elevated levels of catechins, with epigallocatechin-3-gallate (EGCG) assuming particular prominence. Modestly fermented white and yellow teas exhibit slightly diminished catechin levels in relation to green tea, thereby facilitating the emergence of theaflavins and thearubigins [10]. Within semi-fermented oolong tea, catechin oxidation transpires solely at the leaf periphery, thereby positioning itself as intermediate to green tea and black tea. Catechin oxidation in dark tea is mediated by microorganisms, ultimately culminating in the generation of theabrownine. Notwithstanding, a comprehensive chemical profiling of Chinese tea remains an outstanding pursuit. A comprehensive and methodical inquiry underpinned by extensive data analysis is pivotal to obviating ambiguities pertaining to the functional constituents of Chinese teas. The standardization of tea quality holds profound ramifications for both tea enterprises and regulatory oversight.

The classification method based on sensory evaluation has a drawback as it lacks quantitative assessment indicators, which leads to difficulties in tea authentication. In contrast, international standards concentrate more on the physical and chemical attributes of tea. The absence of quantitative indicators poses challenges to tea quality control for producers, consumers, and regulatory agencies. In recent years, various analytical techniques, including thin-layer chromatography [11,12], high-performance liquid chromatography [13,14], and ultra-performance liquid chromatography-tandem mass spectrometry [15,16], have been utilized to determine the chemical composition of tea. However, most of these studies have focused on only a few chemical markers from a small number of teas. Thus, a comprehensive analysis of tea using a single analytical method is still missing [17]. To meet international standards, there is an urgent need for analyzing the major chemical constituents of different types of processed teas. In this research, we established an efficient and rapid UHPLC-DAD method for the determination of 20 components, including catechins, alkaloids, phenolic acids, and flavonols, in 121 tea samples from six distinct types of tea. Furthermore, we identified the potential essential chemical components critical for classifying tea types. By performing a principal component analysis on the chemical composition of the six different types of tea leaves, we conducted a classification analysis of the tea sample characteristics.

## 2. Materials and Methods

### 2.1. Reagents and Materials

Gallic acid (GA), (−)-gallocatechin (GC), caffeine (CAF), theophylline (THEO), (−)-epigallocatechin (EGC), (+)-catechin (C), chlorogenic acid (CHL), theobromine (TB), caffeic acid (CAA), (−)-epicatechin (EC), (−)-epigallocatechin gallate (EGCG), ρ-coumaric acid (COU), (−)-gallocatechin gallate (GCG), ferulic acid (FER), sinapic acid (SIN), epicatechin gallate (ECG), rutin (RUT), myricetin (MYR), quercetin (QUE), and kaempferol (KAE) were purchased from Sigma (St. Louis, MO, USA), and the purity of the reagents was above 95%. The acetonitrile (HPLC grade) was purchased from Merck KgaA (Darmstadt, Germany), and all other reagents including methanol and formic acid were purchased from Sinopharm Chemical Reagent Co., Ltd. (Shanghai, China). Ultrapure water was obtained from a Milli-Q water system (Millipore, Bedford, MA, USA). A total of 121 samples covering six different types of teas, including black tea (BT, *n* = 17), green tea (GT, *n* = 29), yellow tea (YT, *n* = 7), white tea (WT, *n* = 12), oolong tea (OT, *n* = 42), and dark tea (DT, *n* = 14), were purchased from local supermarkets in Beijing and Fuzhou, China, and kept in boxes sealed by tin foil at 4 °C. 

### 2.2. Tea Sample Extraction

The sample extraction method was optimized to enhance the efficiency of extracting key constituents in tea. Tea samples underwent initial drying at 35 °C for 2 h, followed by crushing into powders and passage through a 40 mesh screen (304 stainless steel sieve, Yongkang Jielong Industrial and Trade Co., Ltd., Jinhua, China). The selection of this specific mesh screen aimed to optimize the extraction procedure, achieving elevated dissolution rates while minimizing material loss. An aliquot of 0.5 g sample powder was weighed into an Erlenmeyer flask and 10 mL of the methanol-dimethyl sulfoxide mixture (50:50, *v*/*v*) was added. The mixture was shaken for 15 min at room temperature and centrifuged at 8000 rpm for 15 min at 4 °C. This extraction process was repeated once. The supernatants from the two extracts were combined, diluted to 50 mL with the methanol-dimethyl sulfoxide mixture (50:50, *v*/*v*), and stored at −20 °C until analysis.

### 2.3. Development of UHPLC-DAD Analytical Method

In this study, a UHPLC-DAD system was employed for the analysis. The instrument used for this analysis was the Ultimate 3000 UHPLC (Thermo Fisher Scientific, Milan, Italy). Prior to UHPLC analysis, the extract was filtered through a 0.22 μm microporous membrane, and 1 µL of the filtered extract was injected into the UHPLC system. Chromatographic separation was performed using a reverse phase column (Merck Lichrospher RP-18, 100 mm × 2.1 mm, 2 μm, Hessian, Germany). Mobile phases A and B were 0.1% formic acid and acetonitrile, respectively [18]. The gradient elution procedure was: 0 min, 93% A; 12 min, 80% A; 16 min, 50% A; 20 min, 93% A. The analysis duration for each specimen was 20 min, inclusive of a 4 min column equilibration period. The column temperature and flow rate were maintained at 30 °C and 0.3 mL min^−1^, respectively. For detection, two wavelengths, 280 and 340 nm, were compared in this study using the DAD integrated into the UHPLC system. The developed UHPLC method was validated according to ICH guidelines [18] to ensure fulfillment of current regulatory standards.

### 2.4. Statistical Analysis 

Data were presented as mean ± standard deviation and range (min-max). The differences among different groups (>2 groups) were evaluated using ANOVA adjusted by Tukey post hoc test in SPSS software (SPSS for Windows, Release 19.0, SPSS Inc., Chicago, IL, USA). Different lower cases indicate significant differences (*p* < 0.05). To identify potential chemical biomarkers for the classification of teas, a random forest (RF) algorithm was implemented in the “Random Forest” package [19] under R software (Version 3.5.3, https://www.r-project.org/, accessed on 6 July 2021). RF achieves classification by constructing a series of decision trees. It optimizes the classification by aggregating the inputs across all trees. Although this method cannot be compared to traditional chemometrics, which has a solid statistical foundation, RF possesses several advantages, including excellent predictive capabilities and the ability to balance all variables even in cases of overfitting [20,21]. Principal component analysis (PCA) was used to examine patterns in composition data and to highlight similarities and dissimilarities in the phytochemical contents of the tea products.

## 3. Results 

### 3.1. Development and Validation of a UHPLC-DAD Analytical Method

**Figure 1:**
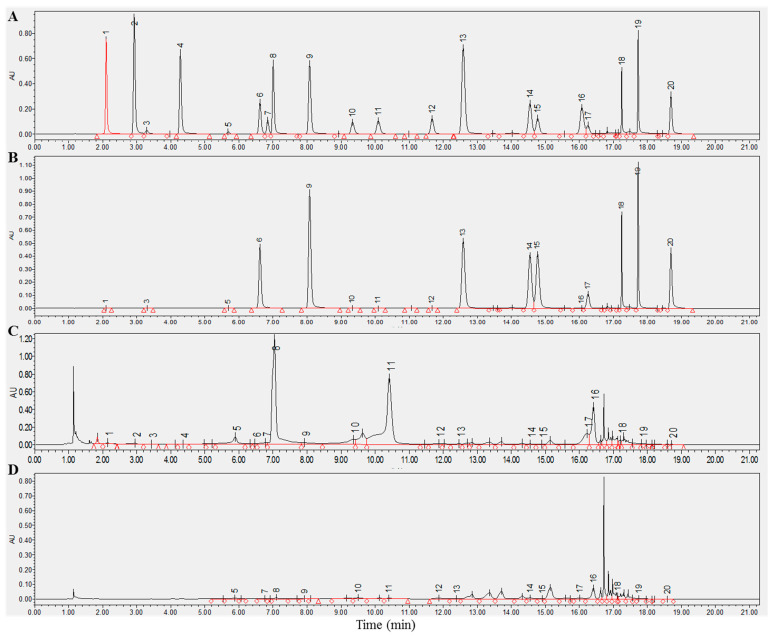
UHPLC-DAD chromatograms of 20 chemical components in tea detected at 280 and 340 nm. (**A**,**B**) A chromatogram of the standard at 280 and 340 nm. (**C**,**D**) A representative chromatogram of the sample at 280 and 340 nm. Component: 1, gallic acid; 2, gallocatechin; 3, caffeine; 4, theophylline; 5, epgallocatechin; 6, catechin; 7, chlorogenic acid; 8, theobromine; 9, caffeic acid; 10, epicatechin; 11, epigallocatechin gallate; 12, coumaric acid; 13, gallocatechin gallate; 14, ferulic acid; 15 sinapic acid; 16, epicatechin gallate; 17, rutin; 18, myricetin; 19, quercetin; 20, kaempferol.

The structures of the 20 compounds are displayed in Figure S2. For enhanced precision, we compared the absorption peaks of these compounds at two wavelengths of 280 and 340 nm (Figure 1B). It came to our attention that all components exhibited enhanced sensitivity and reduced interference at 280 nm, signifying their suitability for concurrent determination of the chosen compounds. We then proceeded to verify the reliability of this analysis method. Table 1 illustrated the favorable linearity of all analytes with R^2^ > 0.999. The relative standard deviations (RSD) were within the range of 0.01–0.31% for intraday assays and 0.42–3.31% for interday assays. Table 1 shows that the limit of detection (LOD) of the analytes was between 0.03 and 2.73 mg/L, alongside the average recovery rate, which ranged from 93.61% to 106.25%. These results indicate that the proposed analysis method was sensitive, precise, and accurate. An analysis of the main chemical components in six different types of teas revealed that green tea contained the highest levels of chemical components (Figure S3).

### 3.2. Comparison of Alkaloids Levels in Six Different Types of Chinese Teas

Our developed method successfully detected three types of alkaloids, namely theophylline (THEO), theobromine (TB), and caffeine (CAF). Remarkably, CAF was the pre-dominant alkaloid in all six types of teas, followed by TB and THEO, indicating that tea processing methods might have had little impact on the alkaloid composition ratio. Nevertheless, our results indicated that different tea types possessed different alkaloid contents. For instance, YT and OT exhibited the highest and lowest TB levels, respectively, while DT showed significantly higher levels of THEO than other tea types (*p* < 0.05, Table 2). Moreover, GT displayed significantly higher levels of CAF compared to OT and WT, and YT exhibited markedly higher TB levels than the other tea types (*p* < 0.05, Table 2). These observations suggested that processing methods may have differentially affected the composition of tea alkaloids, which are known to possess various health-promoting effects.

### 3.3. Dynamic Changes in Catechins in Six Different Types of Tea

Table 3 illustrates the remarkable effect of tea processing methods on the composition ratio of tea catechins. Our data revealed that GC levels were markedly higher in OT and GT compared to other tea varieties (*p* < 0.05, Table 3). Lower EGC levels were detected in DT and BT, while C levels were relatively higher in WT and GT (*p* < 0.05). Notably, WT exhibited significantly higher EC levels than others (*p* < 0.05). The EGCG content was higher in the GT and YT groups, while it was lower in the DT group (*p* < 0.05). Furthermore, the GCG levels in the GT group were significantly higher compared to the WT, DT, and BT groups, and the ECG levels of the GT tea variety were also the highest (*p* < 0.05). Investigating tea catechin changes can aid in regulating tea quality during thermal processing.

### 3.4. Dynamics Changes in Flavonols in Six Different Types of Teas

The tea processing procedures have resulted in a shift in the composition ratio of tea flavonols. As shown in Table 4, OT and WT exhibited a relatively lower proportion of RUT than other tea varieties. WT also contained the lowest level of KAE among all tea types. While BT, GT, and DT demonstrated a higher proportion of QUE, YT had the lowest concentration of QUE. The differences in flavonol levels among the six different tea varieties were not statistically significant (Table 4).

### 3.5. Dynamics Changes in Phenolic Acids in Six Different Types of Tea

In this study, six phenolic acids, namely gallic acid (GA), coumaric acid (COU), chlorogenic acid (CHL), ferulic acid (FER), sinapic acid (SIN), and caffeic acid (CAA) were identified and analyzed. As presented in Table 5, the composition ratio of these components was significantly influenced by tea processing procedures, which classified teas into distinct chemo-types. It is worth noting that CAA was not detected (as nd). DT, BT, WT, and OT exhibited dormancy with GA, while YT displayed dormancy with CHL. OT and GT contained two major phenolic acids.

DT contained a notable quantity of GA, accounting for over 90% of the total phenolic acids, which was significantly higher compared to other tea types (*p* < 0.05, Table 5). A substantial proportion of FER, almost a quarter of the total phenolic acids, was identified in OT and GT (Table 5). Furthermore, the proportion of CHL in GT and YT exceeded that found in other tea types.

### 3.6. Identification of Potential Biomarkers for Tea Classification Using Random Forests 

**Figure 2:**
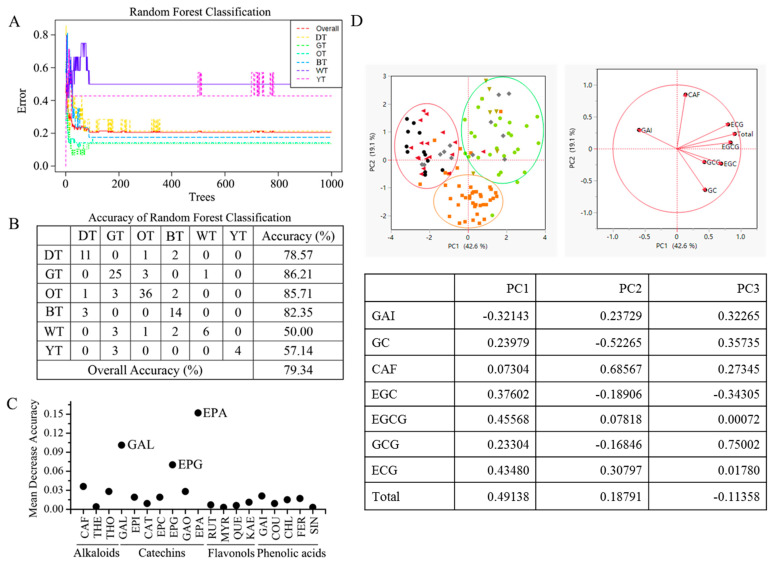
Tea classification based on 19 chemical components using random forests. (**A**) Random forest classification; (**B**) accuracy of random forest classification; (**C**) potential biomarkers identified by random forests. Component: CAF, caffeine; THEO, theophylline; TB, theobromine; GC, (−)-gallocatechin; EGC, (−)-epigallocatechin; C, (+)-catechin; EC, (−)-epicatechin; EGCG, (−)-epigallocatechin gallate; GCG, (−)-gallocatechin gallate; ECG, epicatechin gallate; RUT, rutin; MYR, myricetin; QUE, quercetin; KAE, kaempferol; GA, gallic acid; COU, coumaric acid; CHL, chlorogenic acid; FER, ferulic acid; SIN, sinapic acid. Tea type: BT, black tea; OT, oolong tea; GT, green tea; WT, white tea; DT, dark tea; YT, yellow tea. (**D**) Principal component analysis (PCA) of 6 types of teas. Black: DT, Red: BT, Green: GT, Yellow: YT, Browm: OT, Gray: WT.

In this study, the random forests (RF) classifier was employed to distinguish different types of tea and identify possible biomarkers based on 19 detected chemical components (Figure 2A). The results showed that the proposed RF classifier achieved accuracies of 78.57% for BT, 86.21% for GT, 85.71% for OT, 82.35% for BT, 50.00% for WT, and 57.14% for YT (Figure 2B). The overall accuracy of the classifier was 79.34% using 19 identified chemical components. Although the accuracy of the present classifier was still low, especially for WT and YT, the performance could be improved by increasing the number of samples. In addition, GC, EGCG, and ECG, as the main components of tea catechins, were identified as important biomarkers for tea classification (Figure 2C). Moreover, tea catechins had different responses under thermal processing.

### 3.7. Principal Component Analysis 

Principal component analysis (PCA) was performed on the data obtained from the sample processing of a particular type of tea (Figure 2D). The first principal component (PC1) explained 42.59% of the total variance, while the second principal component (PC2) explained 19.14% of the total variance, and the third principal component (PC3) explained 13.82% of the total variance. Together, PC1, PC2, and PC3 explained a cumulative variance of 75.54%. The results show that the tea samples of six varieties could be classified into three categories based on their fermentation levels. Green tea and yellow tea (non-fermented tea) were categorized together. Oolong tea (semi-fermented tea) was classified separately. Black tea and dark tea (post-fermented tea and fully fermented tea) were grouped together.

## 4. Discussion

Currently, there is a lack of quantitative methods for classifying tea categories in accordance with tea standards. This study aimed to introduce a quantitative and objective approach to identifying tea categories, thereby establishing a scientific foundation for the development of chemical classification methods for tea. Previous research has demonstrated the efficacy of HPLC-DAD in identifying the distinctive constituents of Laoshan green tea (GT) harvested during both summer and autumn, providing accurate determinations of tea leaves across both seasons [22]. Expanding on this foundation, our objective was to formulate and validate a UHPLC-DAD methodology to concurrently quantify 19 key components, encompassing alkaloids, catechins, flavonols, and phenolic acids, within six differently processed teas. Additionally, we endeavored to pinpoint significant biomarkers for tea classification. 

Tea, as a globally consumed beverage, can be classified into six main groups based on the degree of fermentation: green tea (GT), yellow tea (YT), white tea (WT), oolong tea (OT), black tea (BT), and dark tea (DT), in the increasing order of fermentation. The results obtained from our analysis revealed distinct differences in both tea categories and specific compounds as fermentation levels changed. PCA analysis based on these specific compounds also showed good classification results for tea classes with different fermentation levels. These insights contribute to the broader knowledge base surrounding tea, its fermentation process, and its potential implications for health and flavor profiles.

In the present investigation, caffeine was observed to comprise the largest proportion among the six tea types, especially in green tea (GT), which is in accordance with the findings of Boros et al., who extracted caffeine from a variety of teas and reported similar results [23,24]. Interestingly, our results indicated that the processing method for tea leaves had minimal influence on the proportion of alkaloid composition. However, it is worth noting that different alkaloids may react differently to the processing method due to the effects of steeping time, temperature, pH value, and picking time, which all contribute to the chemical composition of tea leaves [25,26,27,28].

Catechins are a standard type of flavonoid found in green tea (GT), and it has been observed that green tea contains more catechins than black or oolong teas [29]. The current study also found that green tea has the highest amount of catechins compared to other tea varieties. Previous studies have indicated that unfermented green tea has EGCG as its main component [29,30], while fermented green tea has GC as its main component with less EGCG [2,5]. Our study confirms that green tea has the highest amount of EGCG, with significantly higher levels than other tea types except for yellow tea. Interestingly, our findings show that DT has the lowest amount of EGCG, which may be attributed to the conversion of catechins to theaflavins or theobromine during the fermentation process in black tea, leading to a significant reduction in total catechins (EGCG, ECG, and EGC) [31,32,33]. Furthermore, different processing methods have been found to affect the ratio of catechin composition in tea leaves, possibly due to the gradual decrease in catechin content and increase in gallic acid content during tea fermentation [5,34]. Another study reported that thermal processing affects the eight catechins in tea differently [35].

Tea flavonols are potent antioxidants that have been shown to protect against cancer and cardiovascular disease [36,37,38], it is of interest to understand the levels of flavonols in different teas and how processing methods impact flavonol composition. We identified RUT, MYR, QUE, and KAE as the major flavonols in tea leaves, in particular RUT and MYR. Our study found that black tea (BT) had lower total flavonol content compared to other teas, which is consistent with the findings of Selim et al. [39,40]. We also found that the gallic acid (GA) content was the highest in black tea (BT) among the 121 tea samples, previous reports indicated that the increase in gallic acid content coupled with deepening fermentation [41,42].

Previous studies classified black, green, white, yellow, dark, and oolong teas by UV spectroscopy [43]; Ding et al., classified tea quality levels based on CLPSO-SVM using near-infrared spectrum [44]. To further explore the potential biomarkers for tea classification, we identified GC, EGCG, and ECG as important biomarkers for tea classification using UHPLC-DAD quantification combined with RF calculations. Previously, Wang et al., identified the physicochemical components such as catechin and caffeine in yellow tea (YT) using quantitative descriptive analysis (QDA) and partial least squares regression (PLSR). RF, although lacking a comprehensive theoretical foundation comparable to traditional chemometrics, offers advantages such as strong predictive capabilities and balanced handling of all variables even in cases of overfitting [20,21]. Additionally, RF’s ability to handle data dimensions without limitations made it a valuable tool for predicting the types of tea accurately. Previous studies have also demonstrated RF’s predictive prowess in tea sample analysis. For example, Zheng et al. [45] demonstrated the superior predictive performance of RF compared to other machine learning methods, such as PCA and SVM, in predicting unknown tea samples. Similarly, Xu et al. [46] used RF based on fused signals to achieve the best performance in predicting the concentrations of chemical components in tea. Our study builds upon this knowledge and showcases the potential of RF in further advancing tea classification methodologies.

In conclusion, we have developed a UHPLC-DAD analytical method to simultaneously determine a total of 19 major components in tea, including alkaloids, catechins, flavonols, and phenolic acids. The method has undergone methodological validation, demonstrating sensitivity, stability, and good repeatability in content determination. Significant differences in these components have been observed among the six types of tea studied, with green tea (GT) and yellow tea (YT) exhibiting higher total chemical content compared to the other teas. These observations suggest that the varying degrees of fermentation in the six major tea categories may influence the composition of alkaloids, catechins, flavanols, and phenolic acids in tea leaves. Furthermore, GC, EGCG, and ECG, serving as the principal constituents of catechins in tea, have been identified as important biomarkers for tea classification. The results of PCA analysis reveal the possibility of categorizing these six tea types into three groups based on their fermentation levels. In future work, we plan to establish a tea composition database and develop a standard analytical method for the evaluation and classification of teas.

## Figures and Tables

**Table 1:** Validation parameters for the UHPLC-DAD method proposed in this study (*n* = 6).

Standard	Calibration Equation	R ^a^	LR ^b^	intraRSD ^c^	interRSD ^d^	LOD ^e^	REC ^f^
GAI	*y* = 19681*x* + 5061.6	0.9998	0.17–100	0.16	1.57	0.05	99.26
GAL	*y* = 17021*x* − 5572.6	0.9998	0.10–100	0.26	0.67	0.03	100.80
CAF	*y* = 15953*x* − 948.43	0.9999	0.63–100	0.21	2.37	0.19	97.44
THE	*y* = 1054.4*x* + 930.46	0.9991	0.33–100	0.31	0.52	0.10	106.25
EPI	*y* = 3799.5*x* + 759.99	0.9999	1.17–100	0.15	0.83	0.35	101.73
CAT	*y* = 8791.6*x* − 2528.8	0.9999	0.60–100	0.13	0.17	0.18	99.20
CHL	*y* = 14512*x* − 426.08	0.9999	0.50–100	0.13	0.42	0.15	95.92
THO	*y* = 20872*x* + 4110	0.9999	0.33–100	0.13	1.28	0.10	98.01
CAA	*y* = 12547*x* + 1496	0.9997	0.43–100	0.13	1.29	0.13	99.71
EPC	*y* = 4710.3*x* + 831.01	0.9998	1.07–100	0.10	0.86	0.32	101.12
EPG	*y* = 7230.3*x* − 3827.3	0.9998	0.87–100	0.15	0.47	0.26	100.01
COU	*y* = 16970*x* + 121844	0.9994	0.63–100	0.17	0.32	0.19	93.61
GAO	*y* = 7167.4*x* − 1372.7	0.9995	0.43–100	0.15	0.68	0.13	94.48
FER	*y* = 17762*x* + 4688.7	0.9999	1.00–100	0.08	0.44	0.30	102.63
SIN	*y* = 6074.3*x* + 1169.5	0.9999	0.37–100	0.06	1.06	0.11	95.24
EPA	*y* = 10336*x* − 2607	0.9999	1.40–100	0.06	0.68	0.42	105.92
RUT	*y* = 2781.4*x* − 1781.5	0.9992	0.40–100	0.08	3.31	0.12	100.17
MYR	*y* = 4158.4*x* − 3148.4	0.9993	0.80–100	0.01	2.53	0.24	96.74
QUE	*y* = 4763.1*x* − 2477.3	0.9997	9.10–100	0.01	3.71	2.73	96.22
KAE	*y* = 7795.4*x* + 5093.3	0.9994	2.17–100	0.01	2.87	0.65	100.95

^a^ correlation coefficient; ^b^ linear range (mg/L); ^c^ relative standard deviation based on intraday assays (%); ^d^ relative standard deviation based on inter-day assays (%); ^e^ limit of detection (mg/L); ^f^ recovery (%). The data presented in mg/L refers to the detection limit of the extraction solution.

**Table 2:** Comparative analysis of alkaloid levels in six different types of teas (*n* = 121), including caffeine (CAF), theophylline (THEO), and theobromine (TB).

Alkaloid	GT (*n* = 29)	YT (*n* = 7)	DT (*n* = 14)	WT (*n* = 12)	OT (*n* = 42)	BT (*n* = 17)	*F* Value	*p* Value
Caffine	53.45 ± 22.07 (0–129.8)	39.40 ± 14.49 (13.90–58.66)	35.94 ± 25.26 (0–103.07)	27.85 ± 23.14 (0.01–63.95) ^a^	25.98 ± 23.49 (0–147.47) ^a^	43.43 ± 29.42 (0–143.89)	3.462	0.006
Percentage of total alkaloids	97.30%	93.84%	95.45%	96.01%	98.03%	97.21%		
Theophylline	0.05 ± 0.05 (0–0.17)	0.07 ± 0.15 (0–0.40)	0.43 ± 0.60 (0–1.63) ^a, b^	0.07 ± 0.10 (0–0.34) ^c^	0.10 ± 0.22 (0–1.25) ^c^	0.06 ± 0.11 (0–0.35) ^c^	5.088	0.000
Percentage of total alkaloids	0.10%	0.17%	1.15%	0.23%	0.29%	0.14%		
Theobromine	1.43 ± 0.69 (0.01–2.50)	2.52 ± 1.67 (0.22–4.75) ^a^	1.28 ± 0.69 (0.25–2.28) ^b^	1.09 ± 0.91 (0.46–3.08) ^b^	0.57 ± 0.46 (0.09–2.36) ^a, b, c^	1.19 ± 0.80 (0.32–2.79) ^b^	10.67	0.000
Percentage of total alkaloids	2.60%	6.00%	3.40%	3.77%	1.68%	2.66%		

Tea type: OT, oolong tea; GT, green tea; WT, white tea; DT, dark tea; YT, yellow tea. Note: Data expressed as mean ± standard deviation and range (min-max). ^a, b, c^ Values with different letters indicate significant differences (*p* < 0.05) compared to GT, YT, and DT samples using ANOVA and Tukey post hoc test. The results were reported in mg/g to indicate the concentration of compounds in 1 g of tea leaves after conversion.

**Table 3:** Comparative analysis of catechin levels in six different types of teas (*n* = 121), including gallocatechin (GC), epicatechin gallate (EGC), epicatechin (EC), epigallocatechin gallate (EGCG), gallocatechin gallate (GCG), epicatechin gallate (ECG), and catechin (C).

Catechin	GT (*n* = 29)	YT (*n* = 7)	DT (*n* = 14)	WT (*n* = 12)	OT (*n* = 42)	BT (*n* = 17)	*F* Value	*p* Value
Gallocatechin	1.55 ± 1.10 (0–4.26)	0.61 ± 0.68 (0–2.00)	0.59 ± 0.29 (0–1.22) ^a^	0.42 ± 0.33 (0–0.98) ^a^	2.31 ± 1.17 (0–4.95) ^a, b, c, d^	0.14 ± 0.21 (0–0.64) ^a, e^	20.70	0.000
Percentage of total catechins	1.10%	0.48%	6.62%	0.39%	2.76%	0.47%		
Epicatechin gallate	22.01 ± 18.94 (0.84–79.73)	14.08 ± 10.57 (4.16–30.84)	1.36 ± 1.20 (0–4.26) ^a^	26.36 ± 32.27 (0.37–95.89) ^c^	20.07 ± 14.00 (0–50.19) ^c^	2.50 ± 2.73 (0–8.7) ^a, d, e^	6.458	0.000
Percentage of total catechins	15.53%	10.98%	15.20%	24.66%	23.95%	8.18%		
Epicatechin	4.50 ± 2.97 (0.67–14.36)	7.02 ± 3.99 (1.14–13.04)	1.14 ± 0.79 (0–2.37)	26.54 ± 36.68 (0.28–84.48) ^a, b, c^	2.62 ± 1.49 (0–6.95) ^d^	0.68 ± 0.89 (0.06–3.40) ^d^	9.478	0.000
Percentage of total catechins	3.18%	5.47%	12.73%	24.82%	3.13%	2.22%		
Epigallocatechin gallate	83.90 ± 31.65 (46.11–158.61)	82.38 ± 14.08 (65.42–107.9)	3.57 ± 5.04 (0.11–18.87) ^a, b^	29.33 ± 30.13 (0.13–78.83) ^a, b^	46.43 ± 19.33 (1.87–86.42) ^a, b, c^	23.85 ± 45.66 (0.05–153.58) ^a, b^	23.42	0.000
Percentage of total catechins	59.21%	64.21%	39.88%	27.43%	55.41%	78.10%		
Gallocatechin gallate	1.16 ± 0.77 (0.14–2.96)	1.15 ± 0.75 (0.32–2.56)	0.32 ± 0.27 (0.03–1.04) ^a^	0.25 ± 0.21 (0–0.55) ^a^	0.89 ± 0.94 (0–3.50)	0.10 ± 0.13 (0.01–0.58) ^a, b, e^	7.591	0.000
Percentage of total catechins	0.82%	0.90%	3.62%	0.23%	1.06%	0.33%		
Epicatechin gallate	24.94 ± 8.42 (11.89–41.84)	20.66 ± 4.02 (15.25–27.86)	1.40 ± 1.12 (0.04–3.55) ^a, b^	18.29 ± 11.45 (3.07–38.27) ^c^	10.09 ± 7.44 (0.44–46.60) ^a, b, c, d^	2.30 ± 1.54 (0.34–4.84) ^a, b, d, e^	34.83	0.000
Percentage of total catechins	17.60%	16.10%	15.64%	17.11%	12.04%	7.53%		
Catechin	3.65 ± 5.83 (0–22.11)	2.39 ± 1.16 (0.78–3.95)	0.57 ± 0.53 (0–1.41)	5.72 ± 15.15 (0.07–53.33)	1.37 ± 2.46 (0–15.31)	0.97 ± 1.19 (0–3.22)	1.809	0.117
Percentage of total catechins	2.57%	1.86%	6.32%	5.35%	1.64%	3.16%		

Tea type: OT, oolong tea; GT, green tea; WT, white tea; DT, dark tea; YT, yellow tea. Note: Data expressed as mean ± standard deviation and range (min-max). ^a, b, c, d, e^ Values with different letters indicate significant differences (*p* < 0.05) compared to GT, YT, DT, WT, and OT samples using ANOVA and Tukey post hoc test. The results were reported in mg/g to indicate the concentration of compounds in 1 g of tea leaves after conversion.

**Table 4:** Comparative analysis of flavonol levels in six different types of teas (*n* = 121), including rutin (RUT), myricetin (MYR), quercetin (QUE), and kaempferol (KAE).

Flavonol	GT (*n* = 29)	YT (*n* = 7)	DT (*n* = 14)	WT (*n* = 12)	OT (*n* = 42)	BT (*n* = 17)	*F* Value	*p* Value
Rutin	2.82 ± 1.49 (0.12–6.91)	1.31 ± 1 (0.28–3.00)	1.52 ± 1.21 (0.18–3.49)	2.29 ± 1.89 (0.38–5.62)	1.86 ± 2.43 (0.15–15.25)	2.34 ± 1.25 (0.31–4.00)	1.586	0.169
Percentage of total flavonols	75.14%	79.54%	77.98%	59.99%	57.68%	74.91%		
Myricetin	0.67 ± 0.78 (0.01–3.74)	0.29 ± 0.24 (0.01–0.66)	0.24 ± 0.25 (0–0.68)	1.46 ± 1.67 (0.04–4.76)	1.22 ± 3.15 (0–14.95)	0.59 ± 0.64 (0.05–2.22)	0.958	0.446
Percentage of total flavonols	17.99%	17.30%	12.30%	38.24%	37.98%	18.76%		
Quercetrin	0.19 ± 0.86 (0–4.66)	0.02 ± 0.01 (0–0.03)	0.13 ± 0.19 (0.01–0.55)	0.06 ± 0.03 (0–0.10)	0.06 ± 0.10 (0–0.66)	0.11 ± 0.12 (0.01–0.50)	0.372	0.866
Percentage of total flavonols	5.00%	1.12%	6.78%	1.45%	2.00%	3.47%		
Kampferol	0.07 ± 0.08 (0–0.25)	0.03 ± 0.03 (0.01–0.07)	0.06 ± 0.11 (0–0.40)	0.01 ± 0.01 (0–0.03)	0.08 ± 0.08 (0–0.34)	0.09 ± 0.12 (0.01–0.39)	1.631	0.157
Percentage of total flavonols	1.87%	2.04%	2.94%	0.31%	2.34%	2.86%		

Tea type: OT, oolong tea; GT, green tea; WT, white tea; DT, dark tea; YT, yellow tea. Note: Data expressed as mean ± standard deviation and range (min-max). ANOVA and Tukey post hoc tests were used to detect no significant differences in the levels of the four flavonols among the six types of tea. The results were reported in mg/g to indicate the concentration of compounds in 1 g of tea leaves after conversion.

**Table 5:** Comparative analysis of phenolic acids levels in six different types of teas (*n* = 121), including gallic acid (GA), chlorogenic acid (CHL), ρ-coumaric acid (COU), ferulic acid (FER), sinapic acid (SIN), and caffeic acid (CAA).

Phenolic Acids	GT (*n* = 29)	YT (*n* = 7)	DT (*n* = 14)	WT (*n* = 12)	OT (*n* = 42)	BT (*n* = 17)	F Value	*p* Value
Gallic acid	0.99 ± 0.52 (0.28–2.51)	1.20 ± 0.66 (0.39–2.03)	4.12 ± 2.89 (1.03–11.57) ^a, b^	2.24 ± 0.87 (0.9–3.61) ^a, c^	1.12 ± 0.94 (0.07–3.71) ^c^	2.54 ± 0.91 (1.23–5.04) ^a, c, e^	16.62	0.000
Percentage of total phenolic acids	24.59%	21.95%	92.72%	10.12%	46.18%	38.70%		
Chlorogenic acid	1.50 ± 4.60 (0–22.83)	3.28 ± 2.61 (0.1–7.88)	0.01 ± 0.03 (0–0.09)	19.25 ± 34.50 (0–80.76)	0.15 ± 0.24 (0–1.29) ^d^	2.79 ± 11.19 (0–46.2)	5.352	0.000
Percentage of total phenolic acids	37.10%	59.93%	0.29%	86.79%	6.26%	42.42%		
ρ-coumaric acid	0.15 ± 0.13 (0–0.46)	0.09 ± 0.11 (0.01–0.32)	0.08 ± 0.18 (0–0.66)	0.12 ± 0.18 (0–0.63)	0.14 ± 0.32 (0–1.64)	0.80 ± 1.64 (0.03–4.97)	3.179	0.010
Percentage of total flavonols	3.82%	1.55%	1.70%	0.54%	5.63%	12.23%		
Ferulic acid	1.05 ± 2.93 (0–15.05)	0.24 ± 0.35 (0.02–1.02)	0.06 ± 0.05 (0.01–0.18)	0.33 ± 0.34 (0.02–0.96)	0.64 ± 0.52 (0–1.62)	0.13 ± 0.13 (0–0.39)	1.358	0.245
Percentage of total phenolic acids	26.16%	4.45%	1.32%	1.47%	26.41%	1.93%		
Sinapic acid	0.34 ± 0.31 (0.03–1.34)	0.66 ± 0.83 (0.2–2.5)	0.18 ± 0.11 (0.04–0.4)	0.24 ± 0.28 (0–0.88)	0.37 ± 0.36 (0–1.32)	0.31 ± 0.46 (0.02–1.88)	1.741	0.131
Percentage of total phenolic acids	8.34%	12.12%	3.96%	1.08%	15.52%	4.72%		
Caffeic acid	nd	nd	nd	nd	nd	nd		

Tea type: OT, oolong tea; GT, green tea; WT, white tea; DT, dark tea; YT, yellow tea. Note: Data expressed as mean ± standard deviation and range (min-max). ^a, b, c, d, e^ Values with different letters indicate significant differences (*p* < 0.05) compared to GT, YT, DT, WT, and OT samples using ANOVA and Tukey post hoc test. The results were reported in mg/g to indicate the concentration of compounds in 1 g of tea leaves after conversion.

## Data Availability

The datasets used and/or analyzed during the current study are available from the corresponding authors upon reasonable request.

## Supplementary Materials

The following supporting information can be downloaded at: https://www.mdpi.com/article/10.3390/foods12163098/s1, Figure S1: Map of China showing the location of all tea sample collection sites; Figure S2: Chemical structures of phenolic acids, alkaloids, flavonol and flavonol glycosides in oolong tea; Figure S3: Changes of major chemical components in six different types of teas.

## Abbreviations

BT: black tea; OT, oolong tea; GT, green tea; WT, white tea; DT, Red tea; YT, yellow tea; CAF, caffeine; THEO, theophylline; TB, theobromine; GC, gallocatechin; EGC, epigallocatechin; C, catechin; EC, epicatechin; EGCG, epigallocatechin gallate; GCG, gallocatechin gallate; ECG, epicatechin gallate; RUT, rutin; MYR, myricetin; QUE, quercetin; KAE, kaempferol; GA, gallic acid; COU, coumaric acid; CHL, chlorogenic acid; FER, ferluic acid; SIN, sinapic acid.
