# Supplementary material for: Quantitative Analysis of Bioactive Compounds in Commercial Teas: Profiling Catechin Alkaloids, Phenolic Acids, and Flavonols Using Targeted Statistical Approaches
Source: Foods. 2023 Aug 17;12(16):3098. doi: 10.3390/foods12163098 (PMC10453493; doi:10.3390/foods12163098)
Supplement: Supplementary file 1 [file foods-12-03098-s001.zip › foods-2464348-supplementary.pdf]

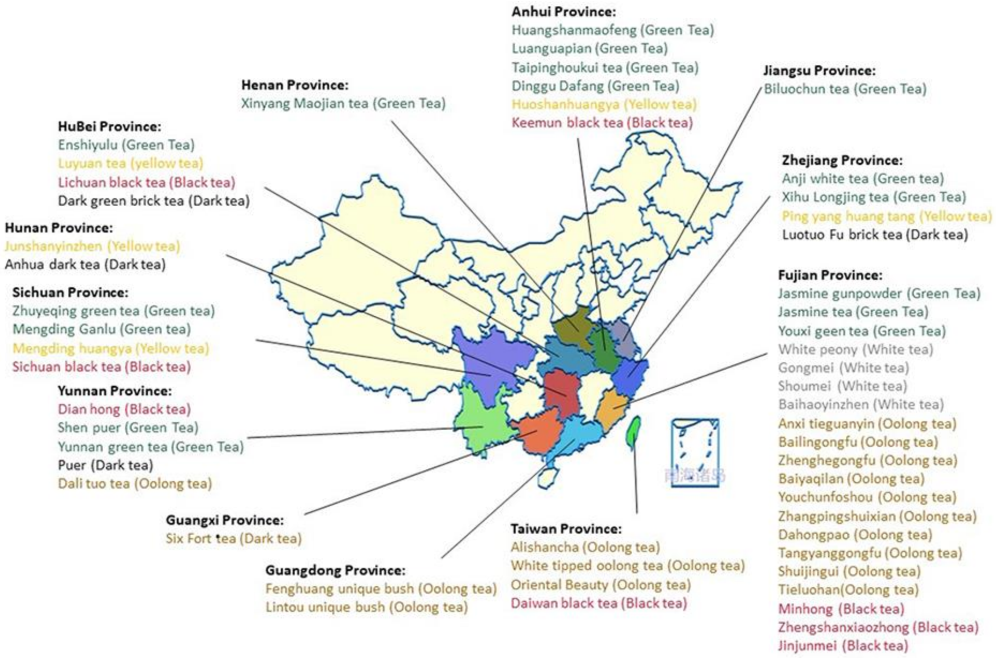

Figure S1. Map of China showing the location of all tea sample collection sites. Abbreviation: GT, green tea extracts; OT, Oolong tea extracts; BT, black tea extract; DT, Dark tea extracts (For interpretation of the references to color in this figure legend, the reader is referred to the web version of this article).

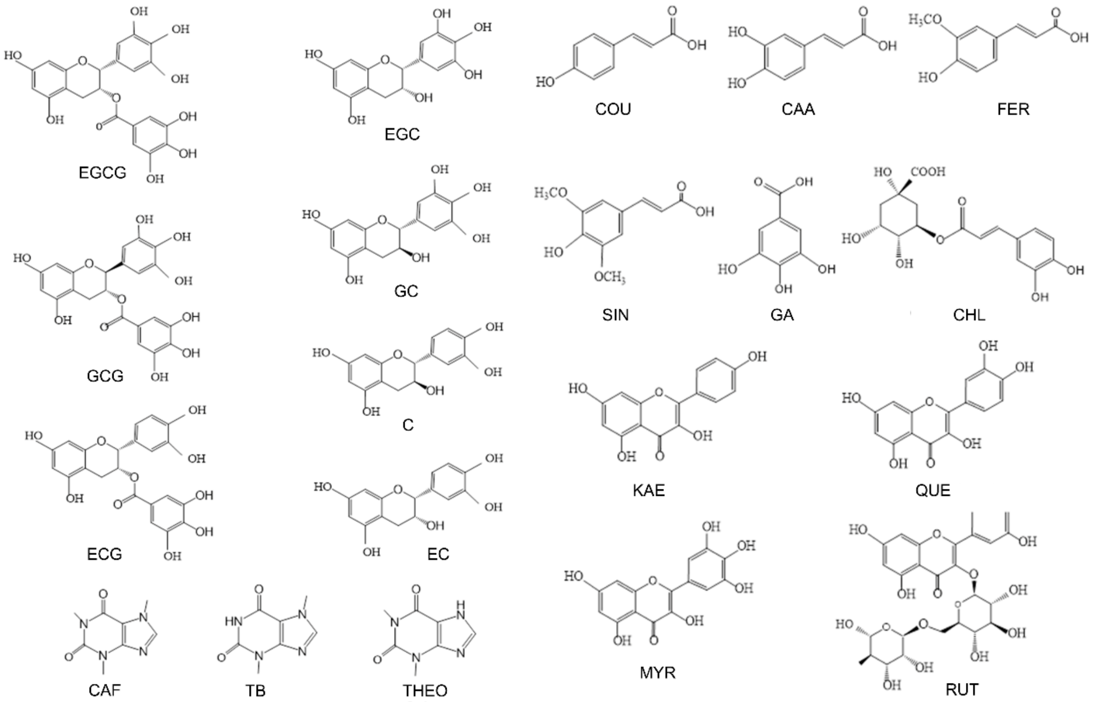

Figure S2. Chemical structures of phenolic acids, alkaloids, flavonol and flavonol glycosides in oolong tea.

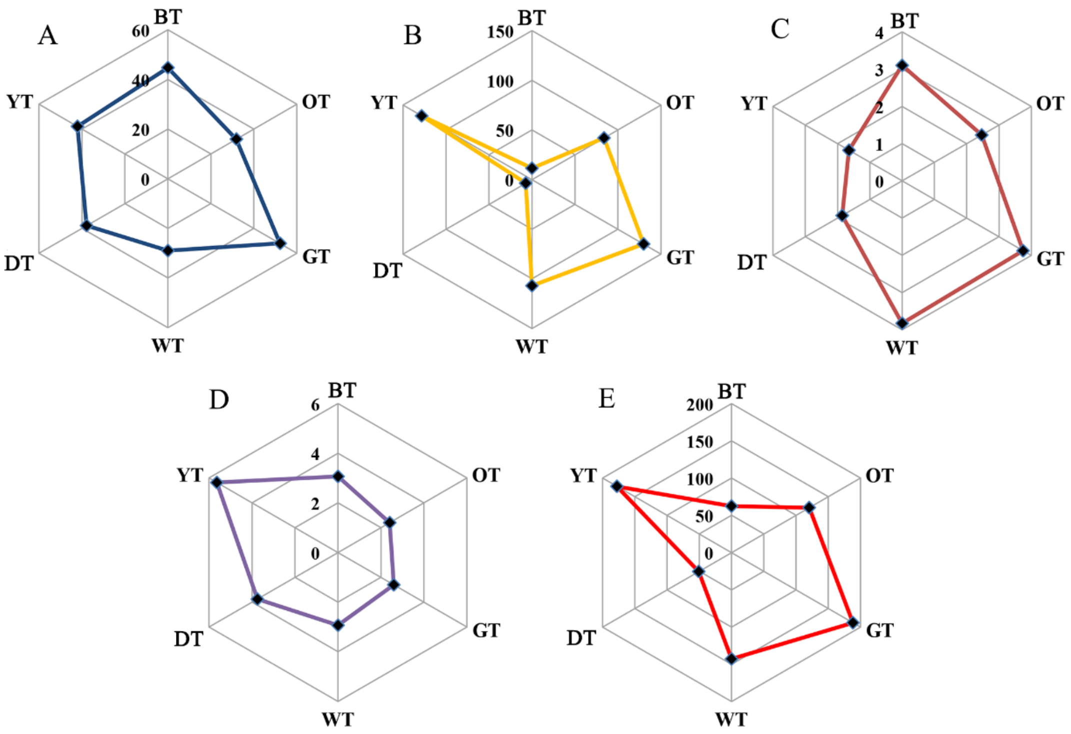

Figure S3. Changes of major chemical components in six different types of teas. (A) Alkaloids; (B) Catechins; (C) Flavonols; (D) Phenolic acids; (E) Total chemical components. Tea type: OT, oolong tea; GT, green tea; WT, white tea; DT, dark tea; YT, yellow tea.
